# Thermodynamics-consistent graph neural networks

**DOI:** 10.1039/d4sc04554h

**Published:** 2024-10-17

**Authors:** Jan G. Rittig, Alexander Mitsos

**Affiliations:** a Process Systems Engineering (AVT.SVT), RWTH Aachen University Forckenbeckstraße 51 52074 Aachen Germany amitsos@alum.mit.edu; b JARA-ENERGY Templergraben 55 52056 Aachen Germany; c Institute of Climate and Energy Systems ICE-1: Energy Systems Engineering, Forschungszentrum Jülich GmbH Wilhelm-Johnen-Straße 52425 Jülich Germany

## Abstract

We propose excess Gibbs free energy graph neural networks (GE-GNNs) for predicting composition-dependent activity coefficients of binary mixtures. The GE-GNN architecture ensures thermodynamic consistency by predicting the molar excess Gibbs free energy and using thermodynamic relations to obtain activity coefficients. As these are differential, automatic differentiation is applied to learn the activity coefficients in an end-to-end manner. Since the architecture is based on fundamental thermodynamics, we do not require additional loss terms to learn thermodynamic consistency. As the output is a fundamental property, we neither impose thermodynamic modeling limitations and assumptions. We demonstrate high accuracy and thermodynamic consistency of the activity coefficient predictions.

## Abstract 2

We propose excess Gibbs free energy graph neural networks (GE-GNNs) for predicting composition-dependent activity coefficients of binary mixtures.

## Graphical abstract

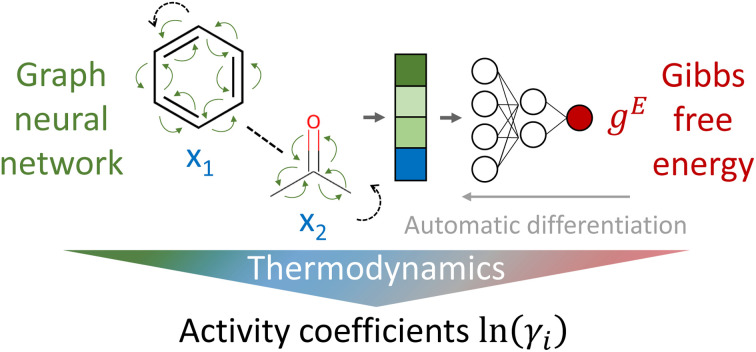

## 1 Introduction

Machine learning (ML) has shown great potential for predicting activity coefficients of binary mixtures which are highly relevant for modeling the nonideal behavior of molecules in mixtures, *e.g.*, in separation processes. Various ML models such as transformers,^1^ graph neural networks (GNNs),^2–7^ and matrix completion methods (MCMs)^8,9^ have been used to predict activity coefficients, exploring different representations of mixtures as strings, graphs, or matrices. These ML models have reached high prediction accuracy beyond well-established thermodynamic models, *cf.* ref. 1,3,8,9, but typically lack thermodynamic consistency.

To include thermodynamic insights, ML has been combined with thermodynamic models in a hybrid fashion, *e.g.*, in ref. 10–14. Hybrid ML models promise higher predictive quality and model interpretability with less required training data. For activity coefficients, ML has been joined with thermodynamic models such as NRTL^15^ and UNIFAC,^16^*cf.* ref. 3,9,17. Since thermodynamic models are associated with theoretical assumptions and corresponding limitations, the resulting hybrid models, however, also exhibit predictive limitations.

We thus recently proposed a physics-informed approach by using thermodynamic consistency equations in model training.^18^ Physics-informed ML uses algebraic and differential relations to the prediction targets in the model architecture and training, and has already been utilized in molecular and materials property prediction, *cf.* ref. 19–22. Specifically for activity coefficients, we added the differential relationship with respect to the composition of the Gibbs–Duhem equation to the loss function of neural network training – in addition to the prediction loss. Due to the high similarities to physics-informed neural networks,^23,24^ we referred to this type of models as Gibbs–Duhem-informed neural networks. The Gibbs–Duhem-informed GNNs and MCMs achieved high prediction accuracy and significantly increased the Gibbs–Duhem consistency of the predictions, compared to models trained on the prediction loss only. However, this approach learns thermodynamic consistency in the form of a regularization term (also referred to as soft constraint) during training. It therefore requires tuning an additional parameter, *i.e.*, weighting factor for the regularization, and does not ensure consistency.

Herein, we propose to instead use thermodynamic differential relationships directly in the activity coefficient prediction step. That is, the output of the ML model is the excess Gibbs free energy, a fundamental thermodynamic property. We then utilize its relationship to the activity coefficients in binary mixtures for making predictions, thereby imposing thermodynamic consistency. Using differential relations to the Gibbs or Helmholtz free energy has already been used in previous studies to develop equations of states with ANNs. For example, Rosenberger *et al.*^20^ and Chaparro & Müller^21^ trained ANNs to predict the Helmholtz free energy with first- and second-order derivatives related to thermophysical properties, such as intensive entropies and heat capacities, by applying automatic differentiation. They could thereby provide thermodynamics-consistent property predictions. However, so far only properties of Lennard-Jones fluids and Mie particles have been considered by using corresponding descriptors, *e.g.*, well depth and attractive/repulsive potentials, as input parameters to an ANN.^20–22^ To cover a diverse set of molecules, we propose to combine thermodynamic differential relations with GNNs. We also extend previous approaches to mixture properties. As a prime example, we combine differential relations of the excess Gibbs free energy with GNNs to predict activity coefficients of a wide spectrum of binary mixtures. During the review process of the current article, Specht *et al.*^25^ proposed a similar approach; more precisely, they utilize the excess Gibbs free energy for activity coefficient prediction with transformer models based on molecular SMILES. Here, we focus on graph-based molecular ML. We call our models excess Gibbs free energy (GE)-GNNs.

## 2 Methods & modeling

**Fig. 1:**
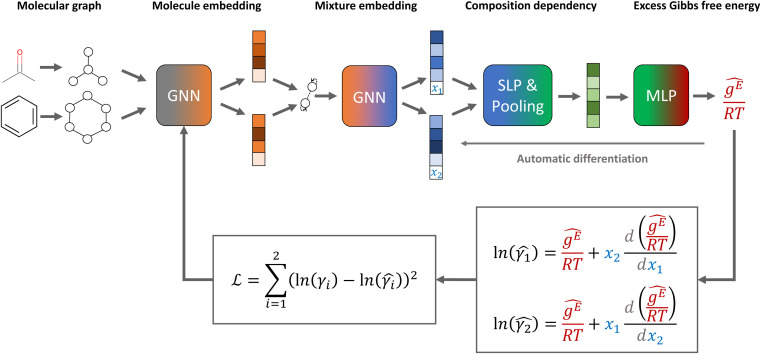
Model structure and loss function of our excess Gibbs free energy graph neural network (GE-GNN) for predicting composition-dependent activity coefficients.

The general architecture of our GE-GNNs is illustrated in Fig. 1. The architecture is inspired by the SolvGNN model proposed by Qin *et al.*,^4^ which we also used for our Gibbs–Duhem-informed GNNs.^18^

### 2.1 Excess Gibbs free energy graph neural networks

The GE-GNN takes molecular graphs as input and first learns molecular vector representations, *i.e.*, molecular fingerprints, in graph convolutions and a pooling step; for details see overviews in ref. 26–31. Then, a mixture graph is constructed with the components being nodes (here two nodes) that have the molecular fingerprints as node feature vectors.^4,6,18^ An additional graph convolutional layer is applied on the mixture graph to capture molecular interactions, resulting in updated molecular fingerprints. We concatenate the compositions to these fingerprints and apply single layer perceptron (SLP) with a subsequent pooling step, yielding a vector representation of the mixture, referred to as mixture fingerprint. Lastly, an MLP takes the mixture fingerprint as input and predicts the molar excess Gibbs free energy.

To obtain activity coefficient predictions, we utilize differential thermodynamic relationships. Specifically, we use the relationship of the activity coefficient in binary mixtures to the molar excess Gibbs free energy (for details see Appendix):1a
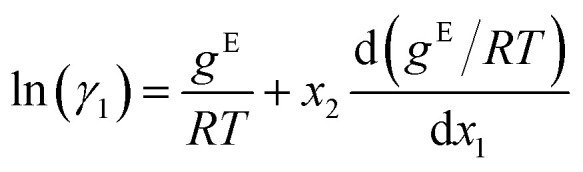
1b
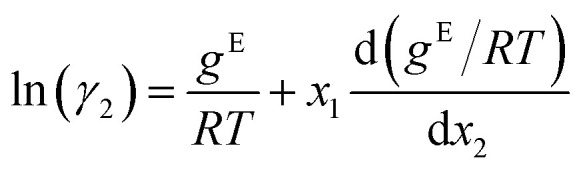


Given eqn (1a) and (1b), we use *g*^E^/*RT* as the prediction target, corresponding to the output node of the GNN, from which we then calculate the binary activity coefficients. The first term of the equations corresponds to the output node, while the second part, *i.e.*, the differential term, can be calculated by using automatic differentiation of the GNN with respect to the compositions. Then, the deviations between the predictions and the (experimental/simulated) activity coefficient data are used in the loss function. Note that since we only consider the activity coefficients at constant temperature (298.15 K), *R* and *T* have constant values and are not considered as additional inputs. So the model is not sensitive to *R* and *T*, and predicting *g*^E^/*RT* translates to predicting *g*^E^. As the Gibbs free energy is a fundamental property, the derived eqn (1a) and (1b) for the activity coefficients are thermodynamically consistent. It is trivial to check that they satisfy for instance the Gibbs–Duhem equation.

To obtain a continuously differentiable prediction curve of the activity coefficient over the composition, which is necessary for thermodynamic consistency, we apply the smooth activation function softplus for the SLP and the MLP. We use softplus as it has been shown to be effective for molecular modeling by Schütt *et al.*^32^ and in our previous work.^18^ Other smooth activation functions could also be used, such as SiLU, for which we found similar performance to softplus. In contrast, using ReLU in the SLP/MLP can cause the model to stop learning in early epochs, resulting in very inaccurate predictions, which is presumably due to the non-smoothness of ReLU. For more details on the effect of the activation function, we refer the interested reader to our previous work.^18^

### 2.2 Mixture permutation invariance

To ensure permutation invariance with respect to the molecular inputs, we express all equations in terms of *x*_1_ (*i.e.*, *x*_2_ = 1 − *x*_1_ and d*x*_1_ = −d*x*_2_) and apply a mean pooling step, in contrast to simply concatenating the two molecular fingerprints, for obtaining the mixture fingerprint. Changing the input order, *e.g.*, ethanol/water *vs.* water/ethanol, thus results in the same activity coefficient predictions for the respective components. We note that the compositions could also be concatenated to the molecular fingerprints before entering the mixture GNN model for modeling molecular interactions, without using an additional SLP to capture the composition dependency. This requires using smooth activation functions (*e.g.*, softplus) in the GNN part to obtain a continuously differentiable activity coefficient curve (*cf.* ref. 18). However, we found this alternative architecture to result in lower prediction performance and higher computational cost, as we have to compute the gradients with respect to the compositions through graph convolution layers, *cf.* our previous work.^18^

### 2.3 Training and evaluation

For training and evaluation, we use the composition-dependent activity coefficient data generated with COSMO-RS^33,34^ by Qin *et al.*^4^ The data set contains 280 000 activity coefficients that correspond to 40 000 binary mixtures based on the combination of 700 different compounds at seven different compositions, specifically {0, 0.1, 0.3, 0.5, 0.7, 0.9, 1}, with 0 and 1 denoting infinite dilution. Analogously to our previous work,^18^ we use different data split types:

In the comp-inter split, activity coefficients at random compositions are excluded for some but not all mixtures, thus testing whether the model learns the composition-dependency of the activity coefficients.

For the comp-extra split, we exclude activity coefficients at specific compositions for all binary mixtures from training and use those for testing, *e.g.*, {0.1, 0.9}. This allows us to assess the generalization capabilities to unseen compositions.

In the mixt-extra split, some binary mixtures are completely excluded from training and the corresponding molecules only occur in other combinations. The excluded mixtures are then used for testing, thereby allowing to evaluate the generalization capabilities to new combinations of molecules.

For comp-inter and mixt-extra, we use a 5-fold stratified split based on polarity features to ensure that all polarity combinations are present in both the training and test sets, analogous to previous studies,^4,18^ whereas for comp-extra all compositions are excluded from training in the respective split. The respective test sets are then used to assess the prediction quality and thermodynamic consistency.

For the predictive quality, we use the root mean squared error (RMSE), the mean absolute error (MAE), and coefficient of determination (*R*^2^) of the predictions and the data. For the thermodynamic consistency, we consider the deviation from the Gibbs–Duhem (GD) differential equation (*cf.* Appendix) in the form of the RMSE, *i.e.*, referred to as GD-RMSE.^18^ The GD-RMSE is evaluated at the compositions of the test data set, *i.e.*, GD-RMSE_test_, and at external compositions for which activity coefficient data are not readily available and are thus not used in training, referred to as GD-RMSE^ext^_test_. Specifically, the external compositions are based on 0.05 steps outside the test set, *i.e.*, *x*^ext^_*i*test _∈ {0.05, 0.15, 0.2, 0.25, 0.35, 0.4, 0.45, 0.55, 0.6, 0.65, 0.75, 0.8, 0.85, 0.95}. In figures, we further consider the MAE for the Gibbs–Duhem differential equation and the molar excess Gibbs free energy.

We provide the code for the model and data splitting as open-source in ref. 35. To ensure comparability to previous models, we use the same model and training hyperparameters as in our previous work.^18^

## 3 Results & discussion

Table 1 shows the prediction accuracy and Gibbs–Duhem consistency for different ML models evaluated on the comp-inter and mixt-extra splits. The SolvGNN by Qin *et al.*^4^ directly predicts activity coefficients; the model is trained on the prediction loss only, *i.e.*, the deviation between predictions and activity coefficient data, without using thermodynamic relations. The GDI-GNN, GDI-GNN_*x*MLP_, and GDI-MCM models are different ML models from our previous work^18^ that also directly predict the activity coefficients and use the Gibbs–Duhem equation as a regularization term in the loss function during training, thereby learning but not imposing thermodynamic consistency. The GDI model training is additionally enhanced by using a data augmentation strategy, that is, the deviation from the Gibbs–Duhem differential relationships at random compositions (not only at the compositions for which activity coefficients are available for training) is also considered in training, so that the models can learn thermodynamic consistency over the whole composition range. We compare these models to the GE-GNN proposed in this work.

**Table 1:** Comparison of prediction accuracy and Gibbs–Duhem consistency for comp-inter and mixt-extra data splits using different machine learning models. Bold print indicates the best performance

Model	Comp-inter			Mixt-extra		
	RMSE_test_	GD-RMSE_test_	GD-RMSE^ext^_test_	RMSE_test_	GD-RMSE_test_	GD-RMSE^ext^_test_
aSolvGNN^4^	0.088	0.212	0.298	0.114	0.206	0.311
GDI-GNN^18^	0.081	0.032	0.038	**0.105**	0.040	0.038
GDI-GNN_*x*MLP_^18^	0.083	0.028	0.025	0.113	0.035	0.030
GDI-MCM^18^	0.088	0.034	0.035	0.120	0.039	0.036
GE-GNN (this work)	**0.068**	**0.000**	**0.000**	0.114	**0.000**	**0.000**

a Model was reevaluated in ref. 18.

The results show that the GE-GNN model outperforms the other models by achieving a higher prediction accuracy based on the RMSE of 0.068 on the comp-inter test set. The GE-GNN further imposes Gibbs–Duhem consistency, *i.e.*, exhibits a GD-RMSE_test_ and a GD-RMSE^ext^_test_ of 0. For the mixt-extra sets, the GDI-GNN shows the highest prediction accuracy in terms of the RMSE of 0.105, whereas the GE-GNN exhibits a slightly worse RMSE of 0.114, but indeed preserves thermodynamic consistency.

**Fig. 2:**
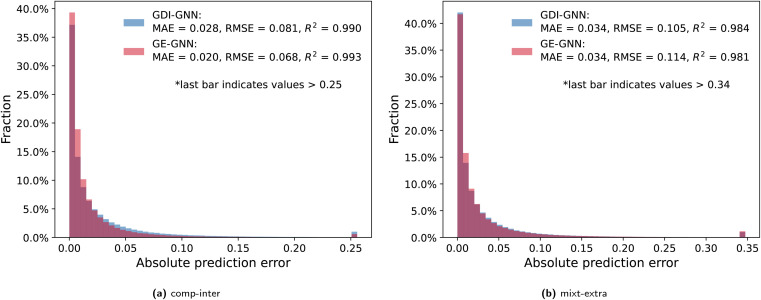
Absolute prediction errors of the GDI-GNN and GE-GNN are illustrated in histograms for the comp-inter (a) and mixt-extra (b) splits. Outlier thresholds are based on the top 1% of the highest errors.

To further analyze the prediction accuracy, we show the distribution of the absolute prediction errors on the comp-inter (a) and mixt-extra (b) splits, respectively, for the two best performing models according to the average prediction RMSE, namely the GDI-GNN and the GE-GNN, in Fig. 2. The error distribution for all models is provided in the Appendix. For the comp-inter split, shown in Fig. 2(a), we find the GE-GNN to have a higher fraction of low prediction errors, that is, 91.0% of the errors are below 0.05 (*vs.* 85.2% by the GDI-GNN). This is also reflected in a lower MAE of 0.020 and higher *R*^2^ of 0.993 compared to a MAE of 0.028 and an *R*^2^ of 0.990 by the GDI-GNN, highlighting the superior prediction accuracy of the GE-GNN for the comp-inter split. For the error distribution of the mixt-extra split, illustrated in Fig. 2(b), we observe that the GE-GNN has a slightly higher fraction of low prediction errors compared to the GDI-GNN, *i.e.*, 84.2% *vs.* 82.9% of the errors are below 0.05. The MAEs for both models are on par, whereas the lower RMSE of the GDI-GNN is also reflected in a slightly higher *R*^2^, which originates from a slightly lower fraction of outliers compared to the GE-GNN; 1% *vs.* 1.15% of the predictions have errors greater than 0.34, respectively. For the mixt-extra split, we thus overall find similar prediction accuracy.

Imposing thermodynamic consistency with respect to the composition therefore seems to have a positive effect on the prediction accuracy for predicting activity coefficients at new compositions, as tested with the comp-inter split. When generalizing to new mixtures (mixt-extra), the structural characteristics of the molecules learned by the GNNs are presumably more important, so that the exact Gibbs–Duhem consistency of the GE-GNN does not result in a significant advantage over the learned consistency by the GDI-GNN in terms of the prediction accuracy. Here, the GE-GNN preserves the high level of accuracy and additionally guarantees thermodynamic consistency.

**Fig. 3:**
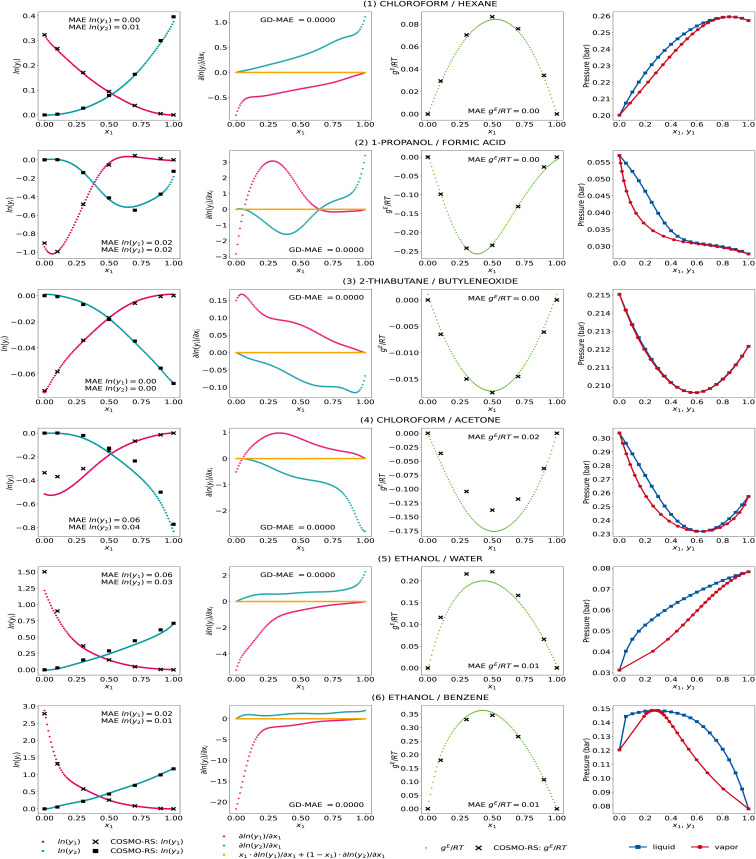
Activity coefficient predictions, their corresponding gradients with respect to the composition with the associated Gibbs–Duhem deviations, the molar excess Gibbs free energy, and vapor–liquid equilibria for exemplary mixtures by the GE-GNN. The predictions are averaged from the five model runs of the comp-inter split, *i.e.*, an ensemble.

We further show the GE-GNN's activity coefficient predictions, the corresponding gradients with respect to the composition, the molar excess Gibbs free energy, and the vapor–liquid-equilibrium (VLE) plots at 298 K for some exemplary mixtures in Fig. 3. We took the same exemplary mixtures as in our previous work on GDI-GNNs (*cf.* ref. 18) to ensure comparability and reflect different nonideal behaviors in binary mixtures, hence different activity coefficient curves. The VLEs are obtained using Raoult's law and the Antoine equation with parameters from the National Institute of Standards and Technology (NIST) Chemistry webbook^36^ based on the work by Qin *et al.* and Contreras.^4,37^

We observe accurate predictions of the activity coefficients that are consistent with the Gibbs–Duhem equation for all mixtures. In particular, for systems (1)–(3) and (6), the predicted activity coefficients match the COSMO-RS data very accurately, which is also reflected in an accurate fit of the molar excess Gibbs free energy. For systems (4) and (5), *i.e.*, chloroform/acetone and ethanol/water, the infinite dilution activity coefficients for the second component (*x*_1_ → 1) show some deviations. For these systems, we also find slight deviations in the activity coefficient predictions at intermediate compositions, which leads to an underestimation of the molar excess Gibbs free energies in both cases. Yet, the general trend in the activity coefficient and corresponding molar excess Gibbs free energies curves is well captured. Furthermore, we observe thermodynamically consistent and smooth VLE plots for all systems, which we have shown to be problematic when ML models are trained only on activity coefficients without using thermodynamic insights, *cf.* ref. 18. The GE-GNNs are therefore able to capture various nonideal behaviors in the exemplary mixtures with thermodynamic consistency and provide overall highly accurate predictions.

In addition, we report the prediction accuracy and thermodynamic consistency for the comp-extra set in Table 2, where we exclude specific compositions for all mixtures from the training set and use them for testing (*cf.* Section 2). We note that this scenario is rather artificial and aims to test the generalization capabilities in an extreme case. In practice, experimental data for these compositions are readily available. We compare the GE-GNN with the same models as for the comp-inter and mixt-extra splits.

**Table 2:** Comparison of prediction accuracy and Gibbs–Duhem consistency for the comp-extra split, *i.e.*, specific compositions excluded from training and used for testing (first row), using different machine learning models. Bold print indicates the best performance^a^

Model	Excl. *x*_*i*_ ∈ {0.5}		Excl. *x*_*i*_ ∈ {0.3, 0.7}		Excl. *x*_*i*_ ∈ {0.1, 0.9}		Excl. *x*_*i*_ ∈ {0, 1}	
	RMSE_test_	GD-RMSE_test_	RMSE_test_	GD-RMSE_test_	RMSE_test_	GD-RMSE_test_	RMSE_test_	GD-RMSE_test_
SolvGNN^4^	0.067	0.453	0.180	1.532	0.302	0.715	0.514	0.101
GDI-GNN^18^	0.040	0.030	0.064	0.034	**0.075**	0.044	0.374	0.026
GDI-GNN_*x*MLP_^18^	0.039	0.021	0.065	0.028	0.087	0.032	**0.332**	0.044
GDI-MCM^18^	0.043	0.039	0.067	0.042	0.094	0.036	0.342	0.051
GE-GNN (this work)	**0.026**	**0.000**	**0.054**	**0.000**	0.085	**0.000**	0.504	**0.000**

a Model was reevaluated in ref. 18.

We observe again that the GE-GNN, being thermodynamically consistent, outperforms the other models in terms of the GD-RMSE_test_. For the accuracy of the predictions, RMSE_test_, we see competitive performance of the GE-GNN for intermediate compositions. For *x*_*i*_ = 0.5 and *x*_*i*_ ∈ {0.3, 0.7}, the GE-GNN shows superior accuracy; for *x*_*i*_ ∈ {0.1, 0.9}, the GDI-GNN performs slightly better. In the case of infinite dilution activity coefficients (*x*_*i*_ ∈ {0, 1}), the GE-GNN is outperformed by the GDI models.

**Fig. 4:**
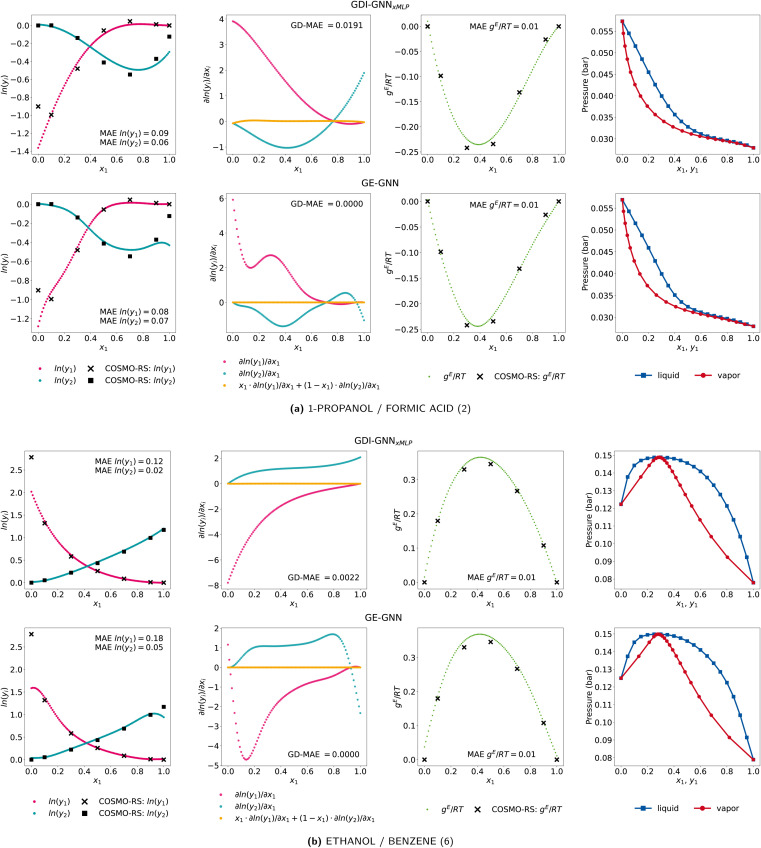
Activity coefficient predictions, their corresponding gradients with respect to the composition with the associated Gibbs–Duhem deviations, the molar excess Gibbs free energy, and vapor–liquid equilibria for the exemplary mixture of (a) 1-propanol/formic acid and (b) ethanol/benzene by the GDI-GNN_*x*MLP_ (top) and GE-GNN (bottom).

To further investigate the lower accuracy of the GE-GNN for infinite dilution activity coefficients, we show two examples of ethanol/benzene and 1-propanol/formic acid of the comp-extra set for both the GDI-GNN_*x*MLP_ and the GE-GNN in Fig. 4. Notably, the slopes of activity coefficients curves predicted by GDI-GNN_*x*MLP_ continue for *x*_*i*_ → {0, 1}. In contrast, the GE-GNN exhibits rather drastic changes in the gradients with respect to compositions in these regions, hence not continuing the slope. We explain this by the fact that the GE-GNN is not trained for these compositions at all and thus cannot interpolate as for intermediate compositions, hence is not sensitive in these regions of extrapolation. The GDI-GNN_*x*MLP_ is trained on Gibbs–Duhem consistency for the whole composition range, *i.e.*, [0, 1], without using any additional activity coefficient data. Thereby, the model seems to learn that having less abrupt variations in the gradients is a way to promote consistency. For binary mixtures, where the infinite dilution activity coefficients can be approximated by a continuation of the nonideal behavior, as for ethanol/benzene, the GDI models yield more accurate predictions. But when binary mixtures exhibit changes in the non-ideal behavior for *x*_*i*_ → {0, 1}, as here 1-propanol/formic acid, both approaches fail to capture these changes, which is expected since they are not trained for these compositions. Therefore, the higher predictive accuracy of the GDI models is presumably due to the fraction of binary mixtures for which the infinite dilution activity coefficients can be approximated by the continuation of the nonideal behavior. As in practice infinite dilution activity coefficients would indeed be utilized for training and it is also possible to include additional data for *x*_*i*_ = 1 with *γ*_*i*_ = 1, *i.e.*, ln(*γ*_*i*_) = 0, the GNNs can learn this non-ideal behavior. Here, it would rather be interesting to extend neural network architectures, including GNNs, to impose this definition of the activity coefficient at *x*_*i*_ = 1, as was recently proposed by Specht *et al.*^25^

## 4 Conclusion

We propose to combine GNNs with thermodynamic differential relationships between properties for binary activity coefficient prediction to ensure thermodynamic consistency. That is, our GE-GNN predicts the excess Gibbs free energy and utilizes the relationship to activity coefficients *via* automatic differentiation during model training, enabling end-to-end learning of activity coefficients. By using a fundamental property as the model output, we do not impose any thermodynamic modeling limitations or assumptions, as opposed to previously proposed ML methods. We further do not need to learn thermodynamic consistency during training, as in physics-informed neural network approaches, which require tuning weighting factors for regularization and do not ensure consistency. Our results show that the GE-GNNs achieve high prediction accuracy and by design exhibit Gibbs–Duhem consistency.

Incorporating additional thermodynamic insights by means of constraining the neural network architecture, *e.g.*, *γ*_*i*_ = 1 for *x*_*i*_ = 1 as in ref. 25, should be addressed in future work. It would also be interesting to capture the temperature-dependency of activity coefficients, *e.g.*, by combining the Gibbs–Helmholtz^6^ with GE-GNNs or directly using the temperature relation in the excess Gibbs free energy.^25^ In general, utilizing further fundamental thermodynamic algebraic/differential relationships is highly promising for future work on combining ML with thermodynamics.

Furthermore, the use of experimental data to train GE-GNNs would be of great practical interest. Here additional challenges will arise, such as experimental noise and uneven distribution of data over compositions and components. Making well-curated experimental activity coefficient data available as open source will remain critical to advancing the field of predictive molecular ML models.

## Data availability

All data and scripts are available as open-source at https://git.rwth-aachen.de/avt-svt/public/GDI-NN.

## Author contributions

J. G. R. developed the concept of excess Gibbs free energy graph neural networks, implemented them, set up and conducted the computational experiments including the formal analysis and visualization, and wrote the original draft of the manuscript. A. M. acquired funding, provided supervision, and edited the manuscript.

## Conflicts of interest

There are no conflicts to declare.

## Appendices

### Relationship of Gibbs free energy and activity coefficients

The relationship between the molar excess Gibbs free energy and the activity coefficients we utilize can be derived from:2
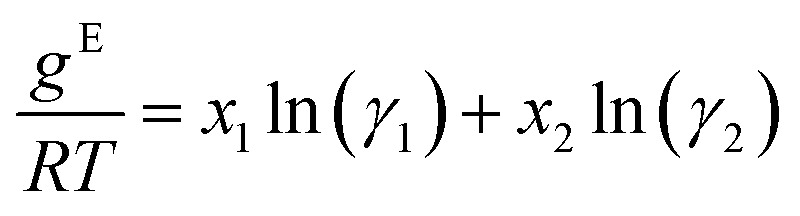


Differentiating eqn (2) with respect to *x*_1_ gives
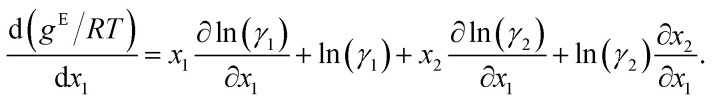


Further inserting the Gibbs–Duhem equation for binary mixtures, *i.e.*,
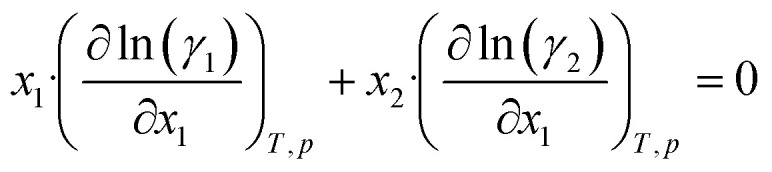


and using d*x*_1_ = −d*x*_2_ yields3
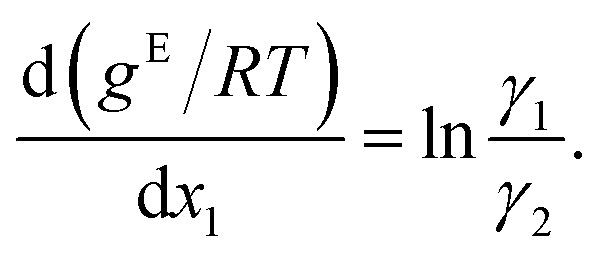


Combining eqn (2) and (3) gives expressions for the binary activity coefficients:
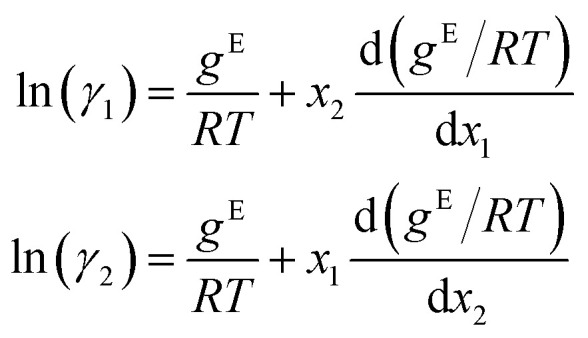


### Additional prediction results

**Fig. 5:**
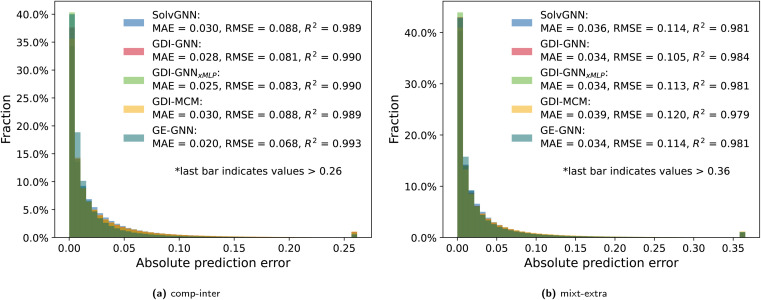
Absolute prediction errors of all models are illustrated in histograms for the comp-inter (a) and mixt-extra (b) splits. Outlier thresholds are based on the top 1% of the highest errors.

Fig. 5 shows the prediction error distributions for the comp-inter and mixt-extra splits for all considered prediction models: SolvGNN, GDI-GNN, GDI-GNN_*x*MLP_, GDI-MCM, and GE-GNN.
